# Prevalence, awareness, treatment and control of diabetes and impaired fasting glucose in the Southern Cone of Latin America

**DOI:** 10.1371/journal.pone.0183953

**Published:** 2017-09-06

**Authors:** Vilma Irazola, Adolfo Rubinstein, Lydia Bazzano, Matias Calandrelli, Chen Chung-Shiuan, Natalia Elorriaga, Laura Gutierrez, Fernando Lanas, Jose A. Manfredi, Nora Mores, Hector Olivera, Rosana Poggio, Jacqueline Ponzo, Pamela Seron, Jiang He

**Affiliations:** 1 South American Center of Excellence for Cardiovascular Health, CESCAS, Institute for Clinical Effectiveness and Health Policy, IECS, Buenos Aires, Argentina; 2 Department of Epidemiology and Department of Medicine, Tulane University School of Public Health and Tropical Medicine, New Orleans, Louisiana, United States of America; 3 Sanatorio San Carlos, San Carlos de Bariloche, Argentina; 4 CIGES, Universidad de La Frontera, Temuco, Chile; 5 Facultad de Medicina, Universidad de la República, Centro Cívico Salvador Allende, Canelones, Uruguay; 6 Municipalidad de Marcos Paz, Buenos Aires, Argentina; West Virginia University School of Medicine, UNITED STATES

## Abstract

**Aims:**

To determine the prevalence, treatment and control of diabetes mellitus (DM) and impaired fasting glucose (IFG) as well as associated factors in the adult population of four cities of the Southern Cone of Latin America (SCLA).

**Methods:**

This is a cross-sectional population-based study that included 7407 adults between 35 and 74 years old in four cities of the SCLA: Temuco (Chile), Marcos Paz and Bariloche (Argentina), and Pando-Barros Blancos (Uruguay). DM was defined as fasting plasma glucose ≥126 mg/dL or self-reported history of diabetes. Awareness, treatment, and control of DM were defined as subjects self-reporting a DM previous diagnosis, the use of a prescription medication or nonpharmacological intervention for DM, and fasting plasma glucose <126 mg/dl, respectively.

**Results:**

Prevalence of DM varied among cities, between 8.4% in Bariloche and 14.3% in Temuco. Prevalence of IFG varied at different sites, from 3.5% in Barros Blancos to 6.8% in Marcos Paz. Of the total number of people with diabetes, 20% were newly diagnosed at the time of the study. Overall, 79.8% of patients with diabetes were aware of their condition. The treatment and control rate were 58.8% and 46.2%, respectively. Older age, family history of diabetes, lower educational attainment, overweight, obesity, central obesity, low physical activity, hypertension, hypercholesterolemia and hypertriglyceridemia were all significantly associated with an increased risk of diabetes.

**Conclusions:**

The prevalence of DM and IFG in the adult population of the SCLA is high and varies among cities. These conditions represent a public health challenge since the rates of awareness, treatment, and control are still low.

## Introduction

An increase in the prevalence of diabetes mellitus (DM) has been recently observed worldwide, in both developed and developing countries. [1–5] According to the International Diabetes Federation (IDF), 8.3% of adults, around 592 million people, suffer from diabetes around the world, and 77% of them live in low and middle income countries (LMIC), where the epidemic is growing at alarming rates. If this trend continues, by 2035 more than 470 million people, that is approximately one adult in 10, will have diabetes. Additionally, since that nearly half of those cases are currently undiagnosed, a huge amount of people with diabetes may be progressing towards complication unaware. [6]

In addition, diabetes is a major risk factor for cardiovascular disease (CVD), the top cause of death in the world [7]. It was estimated that high blood glucose levels accounted for 21% of deaths from ischemic disease, and 13% of all deaths from stroke worldwide. Eighty four percent of these CVD deaths that are attributable to DM occur in LMIC [8].

In Latin America (LA), it is estimated that around 24.1 million people (8.0% of the adult population) suffer from diabetes. [6] As urbanization increases and populations grow older, diabetes may became an even higher priority for the local health systems. In recent years, several population-based surveys have been carried out to determine the prevalence of diabetes in the region, which have contributed substantially to improving the global estimates. However, most of them are based on self-report only, and do not include laboratory measures, which may significantly underestimate the true prevalence of this condition.

The CESCAS I study is a population-based cohort study designed to examine CVD determinants and risk factors prevalence and incidence, and their prospective associations with CVD in four cities of the Southern Cone of Latin America (SCLA). [9] Here, we present the prevalence of diabetes (DM), impaired fasting glucose (IFG) and associated factors from the baseline analysis of the CESCAS I study in order to provide current and reliable data as well as rates of awareness, treatment and control in the adult population between 35 to 74 years old. These results will help inform key stakeholders and policymakers in their combating the challenge related to diabetes in the Southern Cone.

## Material and methods

### Sample design

A detailed description of the study population and design has been presented elsewhere. [10] Briefly, the CESCAS I study used a 4-stage multistage random sample of a general population of 7,524 adults aged 35–74 years from four mid-sized cities in Argentina (Bariloche and Marcos Paz), Chile (Temuco) and Uruguay (Canelones-Barros Blancos). In the first stage, census radii were randomly selected from each of the four locations, stratified by socio-economic level. In the second stage, a number of blocks proportional to the radius size were randomly selected. In the third stage, households within each block were selected by systematic random sampling. All members between 35–74 years in the selected households were listed to create the study sampling frame. In the final stage of sampling, one listed member per household was randomly selected to be included in the study.

The overall response rate was 73.4% and the response rates were similar across different locations. The present analysis was restricted to the 7,407 adults who had Fasting Plasma Glucose (FPG) measured, representing 98.44% of the total population of the CESCAS I Study.

The study protocol was approved by IRBs in all participating institutes in Argentina, Chile, Uruguay and the US, including the Institutional Review Board from Hospital Italiano in Argentina, the Araucanía Sur IRB from the Universidad de la Frontera in Chile, the Universidad de la República IRB from Uruguay, and the Tulane University Human Research Protection Office. All study participants provided written informed consent.

### Data collection and laboratory methods

Study data were collected through standardized questionnaires administered at home by trained interviewers while physical and laboratory measurements were conducted at a clinic visit. During the home survey, information on socio- demographic characteristics and lifestyle factors was obtained, including questions about previous diagnosis, treatment, and family history of DM. Physical activity was measured using the International Physical Activity Questionnaire—Short Form [11]. For each individual, the reported activities were converted into their metabolic equivalent (MET). Low activity was defined as less than 600 MET-minutes/week of total physical activity [12]. Fruit and vegetable intake was measured using a semi-quantitative, self-administered food frequency questionnaire adapted from the NCI Dietary History Questionnaire and validated in Argentina, Chile, and Uruguay [13, 14]. Low fruit and vegetable intake was defined as less than 5 servings per day.

During the clinic visit, blood pressure (BP) and anthropometric measurements were taken by trained and certified examiners using standard protocols. Overnight fasting (≥10 hours) blood specimens were obtained to determine plasma glucose by use of vacuum tubes containing sodium fluoride. Plasma glucose level was measured by using the UV hexokinase enzymatic method (AU5800 Beckman Coulter automatic clinical analyzer). Total cholesterol, HDL-cholesterol, triglycerides, and creatinine were also measured using standard methods. LDL-cholesterol was calculated applying the Friedewald equation for participants who had less than 400 mg/dL triglycerides [15].

DM was defined as FPG ≥126 mg/dL or self-reported history of diabetes [16]. During the home interview, participants were asked if they have ever been told by a doctor or other health professional that they had diabetes (except during pregnancy). Those who answered “yes” to this question were classified as having previously diagnosed DM. Subjects that answered “No” to this question but had FPG ≥126 mg/dL were classified as having unknown DM. IFG was defined as FPG ≥ 110 mg/dL and <126 mg/dL according to WHO diagnostic criteria [WHO]. Awareness of DM was defined as participants’ self-reporting of a previous diagnosis of DM made by a health professional. Treatment of DM was defined as use of prescription medications to lower the blood glucose level, at the time of the home interview. Control of DM was defined as pharmacological treatment of DM associated with FPG <126 mg/dl. We also calculated the overall control rate among all patients suffering from diabetes, including those receiving or not receiving pharmacological treatment.

### Statistical analyses

This study was designed to provide precise estimates of prevalence rates of IFG and DM by gender, age groups (35–44 years, 45–54 years, 55–64 years, and 65–74 years), and city. The sample size was estimated to meet recommended requirements for precision in a complex survey [17]. Prevalence rates were weighted on the basis of the population distribution of the four cities in terms of gender and age according to 2010 census data. [18] Standard errors were calculated taking into account the complex survey design. Age standardized estimates of prevalence were calculated by the direct method, based on the World Standard Population as recommended by the World Health Organization. [19] Logistic regression analysis was used to evaluate the association between socio-demographic, lifestyle, and metabolic risk factors and presence of DM. All data analyses were conducted using SAS 9.3 (SAS Institute, Cary NC) and Stata 13.0 (StataCorp. 2013. College Station, TX: StataCorp LP)

## Results

Characteristics of the study participants according to fasting plasma glucose categories, and stratified by gender are shown in Table 1. A total of 3 103 men and 4 304 women were included. The average age was lower in the normal fasting glucose group in comparison with the other categories for both men and women. The four groups did not differ in the level of educational attainment for men but women showed a greater proportion of lower educational level in the DM groups. Low intake of fruit and vegetables and low physical activity were highly frequent and not significantly different across all categories, with higher levels of low physical activity in women than men. Hypertension, obesity and central obesity were more prevalent among the IFG and DM groups in both men and women.

**Table 1:** Characteristics of study participants according to fasting plasma glucose categories* among men and women aged 35–74 years in the Southern Cone of Latin America*.

	Normal fasting glucose	Impaired fasting glucose	Newly diagnosed diabetes	Previously-diagnosed diabetes
**Men**				
No. (%)	2477 (79.8)	211 (6.8)	117 (3.8)	298 (9.6)
Age (years)	49.5 (49.1, 50.0)	53.2 (51.4, 54.9)	53.1 (50.9, 55.3)	58.8 (57.4, 60.3)
Family history of diabetes (%)	24.1 (22.0, 26.2)	31.0 (23.0, 38.9)	31.2 (20.1, 42.4)	44.7 (38.0, 51.3)
Education				
Primary school (%)	32.8 (30.7, 34.9)	31.4 (24.4, 38.4)	38.0 (27.9, 48.0)	33.6 (27.9, 39.3)
High school (%)	42.0 (39.7, 44.3)	49.0 (40.6, 57.4)	40.9 (29.8, 52.0)	48.4 (41.8, 55.0)
University (%)	25.2 (23.0, 27.4)	19.6 (12.3, 26.9)	21.2 (11.2, 31.1)	17.9 (12.3, 23.5)
Current smoking (%)	35.0 (32.8, 37.3)	24.2 (17.0, 31.4)	29.4 (18.8, 40.1)	21.3 (15.8, 26.7)
Alcohol drinking (%)	59.3 (57.0, 61.6)	57.9 (49.5, 66.2)	53.9 (42.8, 64.9)	53.2 (46.5, 59.9)
Low intake of fruit and vegetables (%)	90.4 (89.1, 91.7)	87.9 (83.1, 92.8)	93.9 (89.7, 98.1)	82.2 (77.4, 87.0)
Low physical Activity (%)	25.0 (23.0, 27.1)	29.7 (21.7, 37.6)	36.4 (25.9, 46.9)	27.2 (21.3, 33.1)
Overweight (%)	49.9 (47.5, 52.2)	36.8 (28.7, 44.9)	34.7 (24.1, 45.3)	41.9 (35.3, 48.4)
Obesity (%)	27.9 (25.8, 29.9)	53.3 (45.0, 61.6)	59.0 (48.2, 69.8)	46.0 (39.4, 52.6)
Central obesity (%)	31.2 (29.1, 33.3)	57.0 (48.7, 65.3)	70.7 (61.3, 80.0)	54.7 (48.1, 61.3)
Hypertension (%)	40.5 (38.3, 42.8)	65.1 (57.1, 73.2)	68.1 (58.1, 78.2)	64.7 (58.1, 71.4)
Hypercholesterolemia (%)	22.1 (20.2, 24.0)	20.2 (14.2, 26.2)	23.3 (14.4, 32.1)	37.4 (30.7, 44.0)
High LDL-cholesterol (%)	21.3 (19.4, 23.1)	17.9 (12.3, 23.5)	21.2 (12.5, 29.9)	33.6 (27.0, 40.1)
Low HDL-cholesterol (%)	45.1 (42.8, 47.4)	54.2 (45.9, 62.4)	51.8 (40.7, 62.8)	55.1 (48.3, 61.8)
Hypertriglyceridemia (%)	26.8 (24.7, 29.0)	45.2 (36.7, 53.6)	64.0 (54.2, 73.8)	33.0 (26.5, 39.5)
**Women**				
No. (%)	3461(80.8)	177(4.1)	107(2.5)	539(12.6)
Age (years)	50.3 (49.9, 50.7)	56.1 (54.0, 58.3)	58.2 (56.2, 60.3)	55.5 (54.3, 56.8)
Family history of diabetes (%)	25.1 (23.2, 27.0)	29.3 (20.5, 38.2)	25.4 (14.9, 35.9)	42.2 (36.9, 47.5)
Education				
Primary school (%)	32.7 (30.9, 34.5)	40.3 (31.5, 49.1)	63.8 (52.1, 75.5)	45.8 (40.6, 51.0)
High school (%)	42.7 (40.6, 44.8)	42.5 (32.7, 52.3)	31.5 (20.0, 43.0)	36.1 (30.9, 41.3)
University (%)	24.6 (22.6, 26.6)	17.2 (9.5, 24.9)	4.7 (0.0, 9.7)	18.1 (13.6, 22.7)
Current smoking (%)	28.3 (26.4, 30.2)	17.3 (8.7, 25.9)	19.6 (10.6, 28.7)	19.0 (14.6, 23.4)
Alcohol drinking (%)	33.1 (31.1, 35.1)	29.7 (21.0, 38.3)	24.8 (14.3, 35.3)	25.1 (20.2, 29.9)
Low intake of fruit and vegetables (%)	81.9 (80.3, 83.5)	74.6 (66.3, 83.0)	83.3 (74.5, 92.0)	80.8 (77.0, 84.7)
Low physical activity (%)	35.8 (33.8, 37.9)	44.1 (34.6, 53.7)	42.4 (30.5, 54.2)	45.1 (39.9, 50.3)
Overweight (%)	36.8 (34.7, 38.8)	31.3 (22.4, 40.3)	16.8 (8.2, 25.4)	31.1 (26.1, 36.1)
Obesity (%)	34.4 (32.4, 36.4)	61.6 (52.4, 70.9)	79.7 (70.8, 88.7)	59.7 (54.5, 64.9)
Central obesity (%)	64.0 (62.0, 66.0)	87.8 (82.2, 93.4)	96.1 (93.0, 99.2)	88.1 (84.6, 91.6)
Hypertension (%)	32.4 (30.6, 34.3)	57.2 (47.4, 67.0)	67.1 (55.9, 78.3)	59.7 (54.4, 65.1)
Hypercholesterolemia (%)	22.6 (21.0, 24.2)	29.3 (21.2, 37.4)	41.6 (30.1, 53.1)	43.2 (37.9, 48.4)
High LDL-cholesterol (%)	21.8 (20.2, 23.5)	23.0 (15.5, 30.6)	32.2 (21.1, 43.3)	40.7 (35.3, 46.0)
Low HDL-cholesterol (%)	21.5 (19.7, 23.4)	31.5 (22.7, 40.2)	35.9 (24.5, 47.4)	28.3 (23.4, 33.3)
Hypertriglyceridemia (%)	13.0 (11.7, 14.4)	22.8 (14.9, 30.6)	36.8 (25.6, 48.0)	27.4 (22.5, 32.2)

Values are percentage or mean (95% confidence interval). To convert the values for glucose to millimoles per liter, multiply by 0.05551. To convert the values for cholesterol to millimoles per liter, multiply by 0.02586. To convert the values for triglycerides to millimoles per liter, multiply by 0.01129. HDL denotes high-density lipoprotein, and LDL low-density lipoprotein.

*Plasma glucose level was categorized as normal fasting glucose (<110 mg per deciliter), impaired fasting glucose (≥110 and <126 mg per deciliter), newly diagnosed diabetes (fasting glucose ≥126 mg per deciliter) and previously-diagnosed diabetes. Low fruit and vegetable intake was defined as <5 servings per day; Low physical activity was defined as <600 MET-minutes/per week; Overweight: body-mass index ≥25 and <30 kg/m^2^; Obesity: body-mass index ≥30 kg/m^2^; Central obesity: waist circumference ≥102 for men and ≥88 cm for women; Hypertension: systolic blood pressure ≥140 mm Hg and/or diastolic blood pressure ≥90 mm Hg and/or use of antihypertensive medication; Hypercholesterolemia: total cholesterol ≥240 mg/dL and/or use of lipid-lowering medication; Low HDL-cholesterol: HDL-cholesterol <40 mg/dL; High LDL-cholesterol: LDL-cholesterol ≥160 mg/dL and/or use of lipid-lowering medication; Hypertriglyceridemia: triglyceride ≥200 mg/dL

Table 2 shows the prevalences of IFG and DM by participant characteristics. The prevalence of DM among the adult population aged 35–74 years old varied between 8.4% in the city of Bariloche and 14.3% in Temuco. The prevalence of IFG varied at different sites, between 3.5% in Barros Blancos and 6.8% in Marcos Paz. Of the total number of people with DM, 20% were newly diagnosed at the time of the study. Prevalence of IFG was higher in men than women (5.9%, 95%CI 5.0–6.9 vs 3.4%, 95%CI 2.7–4.0) while DM was significantly higher in women than men (14.0%, 95%CI 12.8–15.3 vs 10.6%, 95%CI 9.4–11.7). The prevalences of both IFG and DM were increasingly higher with age. The prevalence of DM was higher among individuals with lower education. Prevalences of IFG and DM increased progressively with higher body mass index and waist circumference.

**Table 2:** Estimated prevalence of impaired fasting glucose and diabetes among adults aged 35–74 years in the Southern Cone of Latin America.

	IFG (FPG ≥110 and <126 mg/dl) % (CI 95%)	Newly diagnosed diabetes (FPG ≥126 mg/dl) % (CI 95%)	Previously-diagnosed diabetes % (CI 95%)	Total diabetes % (CI 95%)
Cities				
Marcos Paz, Argentina	6.8 (5.7, 8.0)	4.2 (3.3, 5.2)	7.7 (6.5, 8.9)	11.9 (10.4, 13.4)
Bariloche, Argentina	3.6 (2.8, 4.4)	1.7 (1.1, 2.3)	6.7 (5.6, 7.7)	8.4 (7.2, 9.6)
Temuco, Chile	5.0 (4.0, 6.1)	2.7 (2.0, 3.4)	11.6 (10.1, 13.0)	14.3 (12.7, 15.8)
Barros Blancos, Uruguay	3.5 (2.5, 4.4)	2.1 (1.4, 2.8)	12.1 (10.5, 13.7)	14.2 (12.5, 15.9)
Sex				
Men	5.9 (5.0, 6.9)	3.2 (2.5, 3.9)	7.4 (6.4, 8.3)	10.6 (9.4, 11.7)
Women	3.4 (2.7, 4.0)	1.9 (1.4, 2.3)	12.2 (11.0, 13.4)	14.0 (12.8, 15.3)
Age groups, years				
35–44	3.0 (2.0, 4.0)	1.1 (0.5, 1.7)	5.0 (3.7, 6.2)	6.1 (4.7, 7.4)
45–54	4.3 (3.3, 5.3)	2.9 (2.1, 3.8)	8.2 (6.8, 9.6)	11.1 (9.5, 12.7)
55–64	6.3 (5.2, 7.5)	3.4 (2.5, 4.3)	15.0 (13.2, 16.7)	18.4 (16.5, 20.3)
65–74	6.9 (5.5, 8.3)	4.0 (2.9, 5.1)	20.1 (17.8, 22.3)	24.1 (21.7, 26.4)
Education level				
Primary school	4.7 (3.9, 5.4)	3.5 (2.8, 4.2)	12.1 (10.8, 13.3)	15.6 (14.1, 17.0)
High school	5.0 (4.0, 6.0)	2.2 (1.6, 2.8)	9.5 (8.2, 10.7)	11.7 (10.3, 13.0)
University	3.6 (2.4, 4.8)	1.6 (0.8, 2.3)	7.5 (5.9, 9.2)	9.1 (7.3, 10.9)
Body mass index				
<25.0 kg/m^2^	1.8 (1.0, 2.5)	0.6 (0.3, 0.9)	4.4 (3.4, 5.5)	5.0 (3.9, 6.1)
25.0–29.9 kg/m^2^	3.8 (3.0, 4.7)	1.7 (1.1, 2.2)	8.3 (7.2, 9.5)	10.0 (8.8, 11.2)
≥30.0 kg/m^2^	7.2 (6.1, 8.4)	4.7 (3.8, 5.6)	15.2 (13.6, 16.8)	19.9 (18.1, 21.7)
Waist circumference				
<102 cm in men and 88 cm in women	3.0 (2.3, 3.7)	1.0 (0.7, 1.4)	5.0 (4.2, 5.8)	6.0 (5.1, 6.9)
≥102 cm in men and 88 cm in women	6.0 (5.1, 6.8)	3.8 (3.1, 4.5)	14.3 (13.0, 15.6)	18.1 (16.7, 19.5)

FPG: fasting plasma glucose. To convert plasma glucose to mmol/l, multiply values by 0.0555.

Overall, 79.8% of people individuals with diabetes were aware of their condition, and 73.6% of those aware were receiving antidiabetic medication. Of those receiving pharmacological treatment, 49.2% had FPG <126 mg/dl. The treatment and control rate in the group with DM was 58.8% and 46.2%, respectively (Table 3). Among persons who had reported a prior diagnosis of DM, the overall rate of control was 58.3%. Although the treatment rate did not differ significantly between men and women, both awareness and control rates were higher in women compared to men. Rates of awareness, treatment, and control did not significantly differ across age-groups or educational attainment. The rates of awareness and control were the lowest in Marcos Paz (Argentina) whereas the treatment rate was the lowest in Canelones-Barros Blancos (Uruguay). Awareness and control rates were lower among obese subjects.

**Table 3:** Awareness, treatment and control of diabetes among adults aged 35–74 years in the Southern Cone of Latin America.

	Overall					Men					Women				
	Awareness	Treatment^1^	Treatment^2^	Control^1^	Control^2^	Awareness	Treatment^1^	Treatment^2^	Control^1^	Control^2^	Awareness	Treatment^1^	Treatment^2^	Control^1^	Control^2^
Cities															
Marcos Paz, Argentina	64.5(58.1, 70.8)	77.6(70.9, 84.3)	50.0(43.5, 56.5)	36.9(28.2, 45.6)	27.5(21.6, 33.4)	60.4(50.2, 70.7)	78.2(67.5, 88.9)	47.2(37.0, 57.5)	28.8(15.3, 42.2)	19.8(11.4, 28.2)	68.2(60.7, 75.8)	77.1(68.6, 85.6)	52.6(44.5, 60.7)	43.3(32.3, 54.3)	34.4(26.5, 42.3)
Bariloche, Argentina	78.9(72.6, 85.2)	69.3(61.7, 76.9)	55.1(47.7, 62.5)	45.8(36.1, 55.4)	47.1(39.7, 54.5)	69.4(58.0, 80.8)	78.3(66.5, 90.2)	54.4(42.5, 66.3)	40.7(25.8, 55.6)	34.2(23.0, 45.5)	86.5(80.4, 92.7)	63.5(53.8, 73.2)	55.7(46.5, 64.9)	49.9(37.5, 62.3)	57.5(48.4, 66.7)
Temuco, Chile	81.0(76.5, 85.6)	78.9(73.2, 84.7)	63.9(58.2, 69.7)	51.6(44.3, 59.0)	46.7(40.7, 52.7)	70.2(61.5, 78.9)	84.8(77.4, 92.3)	59.5(50.5, 68.6)	43.8(32.1, 55.5)	33.2(24.6, 41.9)	87.6(83.1, 92.2)	76.0(68.4, 83.7)	66.6(59.2, 74.1)	56.0(46.7, 65.3)	55.3(47.5, 63.0)
Barros Blancos, Uruguay	85.2(80.4, 90.1)	60.9(54.2, 67.6)	51.9(45.5, 58.3)	50.3(41.7, 59.0)	53.3(46.9, 59.7)	74.4(64.3, 84.5)	65.1(53.1, 77.1)	48.4(37.4, 59.5)	33.9(19.0, 48.9)	38.0(27.1, 48.8)	92.0(87.9, 96.1)	58.8(50.7, 66.9)	54.1(46.3, 61.8)	59.2(49.2, 69.2)	62.7(55.3, 70.2)
Age groups, years															
35–44	81.7(73.0, 90.3)	54.3(41.6, 67.1)	44.4(32.9, 55.8)	66.4(49.7, 83.0)	63.0(51.9, 74.1)	52.2(31.9, 72.4)	85.5(68.8, 102)	44.6(24.4, 64.9)	54.4(23.6, 85.3)	31.8(13.0, 50.7)	96.0(92.7, 99.3)	46.1(31.7, 60.5)	44.2(30.3, 58.1)	72.2(52.9, 91.5)	78.5(67.4, 89.6)
45–54	73.7(67.0, 80.3)	70.7(62.8, 78.5)	52.0(44.4, 59.7)	43.7(32.5, 55.0)	39.9(32.3, 47.5)	59.7(47.8, 71.6)	67.4(52.6, 82.2)	40.2(28.2, 52.2)	26.0 (8.4, 43.6)	25.0(14.3, 35.6)	83.175.9, 90.2)	72.2(63.1, 81.3)	60.0(50.6, 69.4)	51.8(38.2, 65.4)	50.3(40.2, 60.3)
55–64	81.3(76.9, 85.8)	79.9(74.9, 84.9)	65.0(59.5, 70.4)	41.6(34.3, 48.8)	39.5(33.8, 45.2)	76.0(68.7, 83.3)	80.5(72.8, 88.3)	61.2(52.9, 69.5)	40.4(29.1, 51.7)	35.6(27.0, 44.1)	85.5(80.0, 91.1)	79.4(73.0, 85.9)	68.0(60.8, 75.1)	42.4(33.0, 51.8)	42.6(34.9, 50.2)
65–74	83.1(78.9, 87.3)	83.0(78.6, 87.4)	69.3(64.1, 74.4)	53.9(46.7, 61.0)	48.3(42.5, 54.1)	81.9(75.3, 88.4)	84.4(78.0, 90.8)	69.1(61.4, 76.8)	43.0(32.4, 53.7)	37.9(29.4, 46.4)	83.9(78.4, 89.4)	82.1(76.2, 88.0)	69.4(62.6, 76.2)	61.6(52.4, 70.8)	55.(48.0, 63.4)
Education level															
Primary School	77.2(73.1, 81.3)	72.0(66.7, 77.3)	55.8(50.8, 60.7)	48.5(41.9, 55.1)	44.2(39.2, 49.2)	67.059.3, 74.8)	74.0(65.5, 82.4)	49.6(41.7, 57.5)	40.0(29.4, 50.7)	30.4(23.1, 37.6)	82.2(77.6, 86.8)	71.2(64.6, 77.7)	58.8(52.6, 65.0)	51.9(43.8, 60.1)	50.9(44.6, 57.2)
Secondary School	81.2(76.3, 86.0)	77.4(71.7, 83.1)	62.8(56.8, 68.8)	43.7(35.8, 51.7)	43.1(36.8, 49.3)	73.1(64.8, 81.4)	82.9(75.6, 90.2)	60.6(51.8, 69.4)	34.5(23.7, 45.3)	31.5(23.3, 39.7)	88.2(83.1, 93.3)	73.4(65.3, 81.6)	64.8(56.7, 72.9)	51.6(40.4, 62.7)	53.8(45.0, 62.5)
University	83.0(75.3, 90.6)	69.3(58.5, 80.0)	57.5(47.3, 67.6)	65.0(53.0, 77.0)	58.0(47.9, 68.1)	66.0(51.5, 80.6)	79.5(65.4, 93.6)	52.5(37.6, 67.4)	57.7(38.0, 77.3)	40.4(25.3, 55.4)	96.2(92.0, 100)	63.8(49.5, 78.0)	61.3(47.4, 75.2)	69.8(54.6, 84.9)	71.5(59.2, 83.7)
Body mass index															
<25.0 kg/m2	88.6(82.6, 94.7)	52.0(40.0, 64.1)	46.1(35.1, 57.1)	54.2(38.7, 69.7)	61.4(50.8, 71.9)	81.6(69.8, 93.4)	65.9(46.9, 84.9)	53.8(37.1, 70.5)	46.4(24.6, 68.1)	45.7(28.7, 62.7)	94.5(89.8, 99.2)	42.0(27.0, 57.0)	39.7(25.5, 54.0)	63.7(42.4, 85.0)	75.4(63.8, 87.0)
25.0–29.9 kg/m2	83.3(78.5, 88.2)	74.6(68.1, 81.0)	62.1(55.7, 68.5)	49.9(41.5, 58.3)	48.3(41.6, 54.9)	73.5(65.0, 81.9)	82.9(75.5, 90.3)	60.9(51.9, 69.9)	37.3(25.9, 48.7)	31.6(23.2, 40.0)	92.4(88.2, 96.5)	68.5(59.1, 77.9)	63.3(54.2, 72.3)	61.0(49.9, 72.1)	63.6(54.8, 72.4)
≥30.0 kg/m2	76.2(72.1, 80.4)	77.1(72.3, 81.9)	58.9(54.1, 63.8)	48.2(41.7, 54.7)	42.6(37.6, 47.5)	64.1(56.1, 72.1)	79.5(72.2, 86.9)	51.0(42.8, 59.1)	41.5(30.2, 52.9)	31.0(23.4, 38.6)	82.8(78.6, 87.1)	76.1(70.0, 82.2)	63.2(57.3, 69.1)	51.2(43.3, 59.1)	49.0(42.8, 55.2)
Waist circumference															
<102 cm in men and 88 cm in women	82.9(77.6, 88.2)	67.6(59.4, 75.7)	56.0(48.5, 63.5)	50.1(40.2, 60.1)	49.3(41.7, 56.9)	78.0(71.0, 85.0)	78.8(70.5, 87.2)	61.5(53.1, 69.9)	40.0(29.1, 51.0)	35.1(26.6, 43.5)	95.2(91.3, 99.1)	44.3(28.9, 59.6)	42.1(27.5, 56.7)	88.4(77.7, 99.2)	87.2(80.0, 94.5)
≥102 cm in men and 88 cm in women	78.9(75.4, 82.3)	75.5(71.3, 79.7)	59.7(55.5, 63.8)	48.9(43.3, 54.4)	45.2(40.9, 49.5)	64.0(56.4, 71.5)	79.6(73.0, 86.3)	50.9(43.4, 58.5)	40.4(29.9, 51.0)	31.2(24.2, 38.2)	85.6(82.3, 88.9)	74.1(69.0, 79.2)	63.6(58.7, 68.5)	51.9(45.3, 58.4)	51.6(46.4, 56.7)

Data are weighted percentages (95% confidence intervals).

Awareness was defined as the proportion of individuals who reported a history of physician-diagnosed diabetes among all diabetes patients. Treatment^1^ and Treatment^2^ were defined as the proportion of individuals using anti-diabetic medications among aware and total patients with diabetes, respectively. Control^1^ was defined as the proportion of individuals with a fasting plasma glucose <126 mg/dL among patients with diabetes who were treated with anti-diabetic medications. Control^2^ was defined as fasting plasma glucose <126 mg/dL among all individuals with diabetes.

In the multivariable analysis, older age, family history of diabetes, lower educational attainment, overweight, obesity, central obesity, low physical activity, hypertension, hypercholesterolemia and hypertriglyceridemia were all significantly associated with an increased risk of diabetes (Table 4). Additionally, the association with male gender was marginally significant.

**Table 4:** Multivariable adjusted risk factors for diabetes among adults aged 35–74 years in the Southern Cone of Latin America.

Risk factors	Diabetes	
	Odds ratio (95% CI)	P value
Male sex	1.12 (0.92, 1.36)	0.07
Age (per 5-year increment)	1.25 (1.19, 1.31)	p<0.0001
Parental history of diabetes	2.5 (2.07, 3.04)	p<0.0001
Primary school vs college	1.34 (1.03, 1.74)	0.03
High school vs college	1.21 (0.92, 1.58)	0.18
Overweight	1.4 (1.04, 1.87)	0.03
Obesity	2.33 (1.66, 3.27)	p<0.0001
Central obesity	1.68 (1.28, 2.19)	p<0.0001
Low physical activity	1.24 (1.04, 1.49)	0.002
Hypertension	1.51 (1.24, 1.83)	p<0.0001
Hypercholesterolemia	1.51 (1.25, 1.83)	p<0.0001
HyperTriglyceridemia	1.63 (1.34, 1.98)	p<0.0001

Low physical activity was defined as <600 MET-minutes/per week; Overweight: body-mass index ≥25 and <30 kg/m^2^; Obesity: body-mass index ≥30 kg/m^2^; Central obesity: waist circumference ≥102 for men and ≥88 cm for women; Hypertension: systolic blood pressure ≥140 mm Hg and/or diastolic blood pressure ≥90 mm Hg and/or use of antihypertensive medication; Hypercholesterolemia: total cholesterol ≥240 mg/dL and/or use of lipid-lowering medication; Hypertriglyceridemia: triglyceride ≥200 mg/dL

CI: confidence interval. Current smoking and Alcohol consumption were tested in the model (p 0.17 and 0.16, respectively).

**Fig 1:**
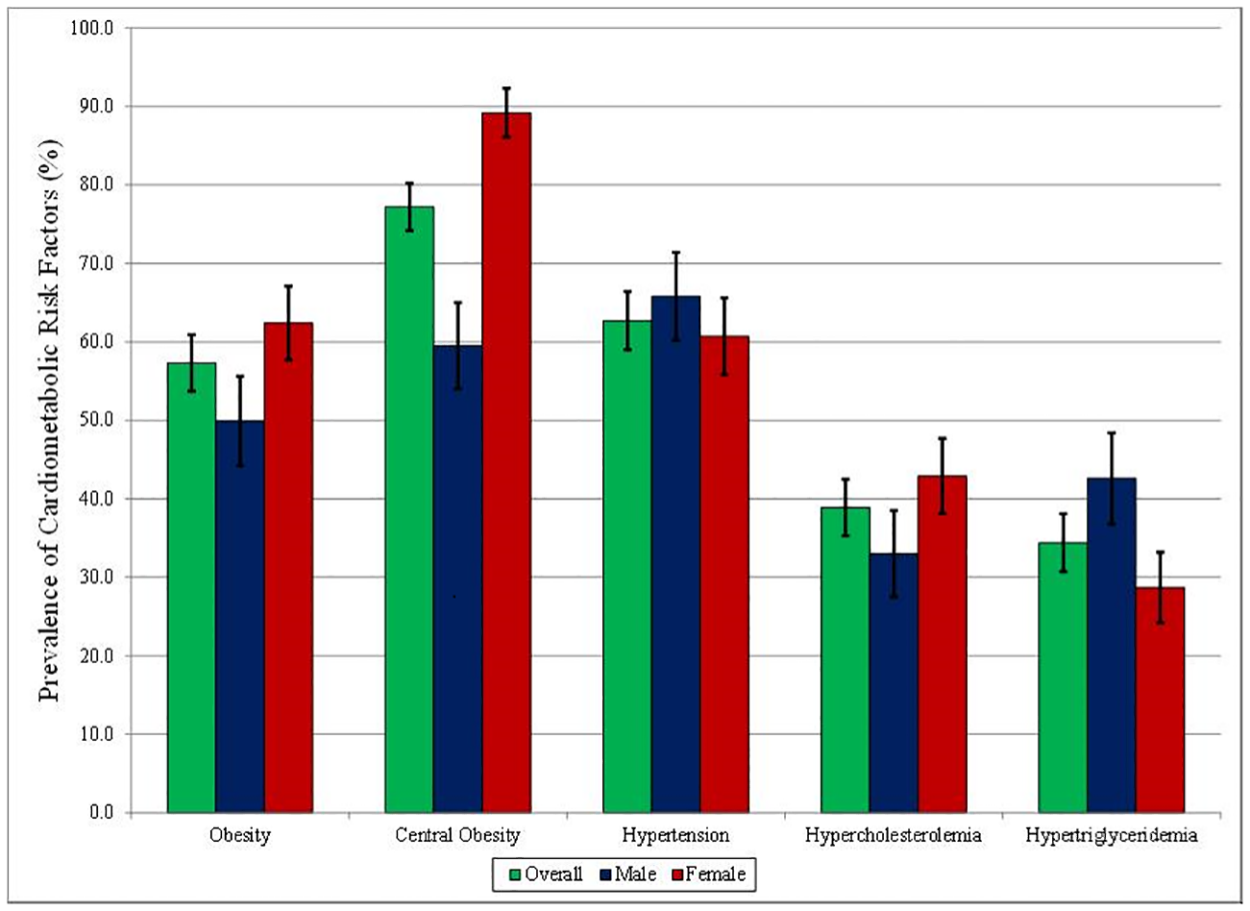
Cardio-metabolic risk factors in subjects with diabetes among adults aged 35–74 years in the Southern Cone of Latin America. Vertical lines indicate 95% confidence intervals.

A high prevalence of other cardiometabolic risk factors was found in patients suffering from DM. As shown in Fig 1, 57.3% of individuals with diabetes were obese, 77.2% had central obesity, 62.7% were hypertensive, 38.9% had hypercholesterolemia and 34.4% had hypertriglyceridemia.

## Discussion

Our results indicate that the prevalence of DM in different cities of the Southern Cone of Latin America goes from 8.4% in Bariloche (Argentina) to 11.9%, 14.2% and 14.3% in Marcos Paz (Argentina), Barros Blancos (Uruguay) and Temuco (Chile) respectively, in adults between 35 and 74 years of age. Almost 20% of these cases were undiagnosed. The prevalence of IFG reached 3.5%, 3.6%, 5.0% and 6.8% in Barros Blancos, Bariloche, Temuco and Marcos Paz, respectively.

IFG and diabetes are rising globally as a consequence of population ageing, urbanization, industrialization, and changes in lifestyle, including diet and physical activity. [1–6] Although a few epidemiological studies of DM have been conducted in Latin America in recent years, data on awareness, treatment, and control are scarce since most of these studies do not include laboratory measurements. Precise and reliable information about the prevalence, awareness, treatment, and control of DM are needed for evidence-informed decision making in public health to combat this epidemic. To our knowledge, these are the first results from a population-based study in this decade in the region with reliable estimates from blood measurements.

Previous national surveys or regional studies have documented a rapid increase in the prevalence of DM in the adult population in the region [5, 7, 20–26]. In Argentina, the National Risk factor Survey conducted in 2013 showed a prevalence of DM of 9.8% in contrast to 9.6% in 2009 and 8.4% in 2005. [20–23] However, these surveys included people 18 years and older, and were based on self-report only, which may have contributed to some underestimation of the true prevalence. The Health National Survey in Chile and the national Risk Factor Survey in Uruguay reported a prevalence of DM of 9.4% and 7.8% in 2010 and 2013 respectively. [24–26] Both surveys included people aged 15 years and older, and while the Chilean survey had blood samples, the Uruguayan survey took blood specimens only in 54% of the sample population. Ten years ago, the CARMELA study had reported a DM prevalence of 6.2% in the city of Buenos Aires, and 7.2% in Santiago de Chile. [27] In other countries of Latin America, the prevalence of diabetes varies widely. [6, 28, 29] However, comparisons between countries should be carried out with caution due to differences in the methodologies employed.

In our population, the prevalence of DM varied highly between cities. This finding is in accordance with the important variability observed among different areas in Argentina in the National Risk Factors Survey (2009), where the prevalence of DM was 7.4% (95% CI 5.9–9.4%) in the province of Jujuy in the north of the country and 13.0% (95% CI 11.0–15.4%) in the province of Cordoba, in the central region. [21] In the same way, the Health National Survey in Chile reported a prevalence of DM of 7.4% (95% CI 7.7–20.2%) in the region of Araucania while 5.1% (95% CI 3.1–8.2%) in the region of Maule. Of note, distribution by gender, age and education was the same in the four cities included in our study.

In our population, DM was more prevalent in women (14.0%) than in men (10.6%) while IFG was the opposite (3.4% in women and 5.9% in men) in accordance with other studies. [30]

The proportion of women with unknown DM was less, compared to men. This sex difference can be explained by the fact that men may have less contact with the health care system than women [31]. We also observed a higher prevalence of both diabetes and IFG with increasing age.

Of note, there was a gradient in the prevalence of DM according to educational level, being higher among subjects with lower educational attainment. This inverse relationship between education and prevalence of diabetes had been reported previously in different populations. [32, 33]

Finally, both DM and IFG were more prevalent in subjects with overweight, obesity and central obesity. In the same venue, the prevalence of dyslipidemia was higher among persons with DM compared to the group without DM, showing the cardio-metabolic nature of this disorder. [34]

Importantly, approximately 1 out of 4 persons with diabetes were not aware of their condition. Of those aware, 73.6% were receiving pharmacological treatment, but only nearly half of those who were treated had their blood glucose controlled. Thus, at the end of the day, the overall control rate among the total number of persons with DM was 46.2%.

In our study, several factors were independently associated with DM since the likelihood of having this condition was increased with increasing age, family history of diabetes, low educational attainment, overweight, obesity, central obesity, low physical activity, hypertension, hypercholesterolemia and hypertriglyceridemia, while the association with male gender was marginally significant. Consistently, these factors were found to be associated with the presence of DM in the four cities included in the study in spite of differences in the local prevalence rate.

Finally, more than half of subjects with diabetes also suffered from hypertension and/or obesity while almost 40% had dyslipidemia. DM, hypertension and dyslipidemia are common and frequently coexisting conditions that share a significant overlap in underlying risk factors, including lifestyle determinants [33, 34]. This joint lifestyle factors provide the opportunity to work on nonpharmacologic interventions such as weight control, physical activity, and dietary modification, which may greatly benefit individuals with comorbidities [35]. Additionally, the presence of both hypertension and DM, double the risk for cardiovascular disease, hence emphasizing its relevance for public health, and the need for prevention, early recognition and adequate treatment of these conditions. [36]

The present study has several strengths. First, this is a population-based study that used a multistage sampling method to select a representative sample of the general population aged 35–74 years in four cities of the Southern Cone of Latin America. Second, all measurements followed a rigorous protocol and used standardized questionnaires applied by trained interviewers. Third, plasma determinations were carried out following standardized procedures of blood-sampling technique and processing, as well as safe transportation of blood samples. Fourth, all blood determinations were centralized and analyzed using the same standardized protocol. All these features enhance the accuracy of prevalence estimations. Additionally, since the CESCAS I study has been planned as a cohort study with long-term follow-up, we will be able to detect changes over time.

It is a limitation of this study that FPG was used for diagnosis of IFG instead of the oral glucose tolerance test (OGTT) since this may have had implications for the population prevalence of prediabetes. It is likely that should OGTT been measured, the prevalence of prediabetes may have been higher. [30] Additionally, FPG, not Hemoglobin A1c, was used as the indicator for DM control, which may have affected the estimated control rate. [30, 36–39] However, FPG is still considered a good standard for epidemiological studies in terms of its lower cost, easier determination, as well as the high correlation between these measures. [5, 40, 41] Another limitation of this study is that the sampling frame in each country is not nationally representative. Although study samples were randomly selected from each city included, caution is needed to extrapolate our findings to each country in the region. Nevertheless, our findings are consistent with the results shown in national surveys of the Southern Cone, which suggests no major biases due to the selection of cities included in the CESCAS I Study. Moreover, the socio-demographic distribution of the population of those cities included in this study are comparable to the national population distribution in each country. Finally, in spite of differences in the prevalence of diabetes among cities, risk factors associated with this condition in multivariable models were consistent across locations.

In summary, the present study indicates that the prevalence of DM and IFG in the adult population of the SCLA is high, and may be underestimated by local surveys that are based only on self-report. This condition represents a public health challenge since the rates of awareness, treatment, and control are still low. Data from this study also support the association of obesity, central obesity, hypertension, elevated serum triglycerides and elevated total cholesterol with diabetes in our population, and indicate that complex intersectoral, integrated and multifaceted interventions, programs and policies should be targeted to counter these cardiometabolic risk factors in order to reduce the burden of NCDs in the region.

## Supporting information

S1 File Diabetes in the Southern Cone Database. (XLSX)

## References

[1] Global status report on non-communicable diseases 2014. Geneva, World Health Organization, 2014.

[2] World Health Organization. Global Health Estimates: Deaths by Cause, Age, Sex and Country, 2000–2012. Geneva, WHO, 2014.

[3] Mathers CD, Loncar D. Projections of global mortality and burden of disease from 2002 to 2030. PLoS Med, 2006, 3(11):e442 doi: 10.1371/journal.pmed.0030442 17132052 10.1371/journal.pmed.0030442 PMC1664601

[4] Lim S., Vos T., Flaxman A. D. et al, A comparative risk assessment of burden of disease and injury attributable to 67 risk factors and risk factor clusters in 21 regions, 1990–2010: a systematic analysis for the Global Burden of Disease study 2010. Lancet 2012; 380: 2224–2260. doi: 10.1016/S0140-6736(12)61766-8 23245609 10.1016/S0140-6736(12)61766-8 PMC4156511

[5] Danaei G, Finucane MM, Lu Y, et al National, regional, and global trends in fasting plasma glucose and diabetes prevalence since 1980: systematic analysis of health examination surveys and epidemiological studies with 370 country-years and 2・7 million participants. Lancet 2011;378:31–40. doi: 10.1016/S0140-6736(11)60679-X 21705069 10.1016/S0140-6736(11)60679-X

[6] Aguiree, Florencia, Brown, Alex, Cho, Nam Ho, Dahlquist, Gisela, Dodd, Sheree, Dunning, Trisha, et al. IDF Diabetes Atlas 2014 Update: sixth edition, 6th ed., International Diabetes Federation, Basel, Switzerland. http://www.idf.org/diabetesatlas. Last access October 1, 2015.

[7] Cardiovascular disease, chronic kidney disease, and diabetes mortality burden of cardiometabolic risk factors from 1980 to 2010: a comparative risk assessment. Global Burden of Metabolic Risk Factors for Chronic Diseases Collaboration. Lancet Diabetes Endocrinol. 2014 8;2(8):634–47. doi: 10.1016/S2213-8587(14)70102-0 24842598 10.1016/S2213-8587(14)70102-0 PMC4572741

[8] Danaei G, Lawes CM, Vander Hoorn S, Murray CJ, Ezzati M. Global and regional mortality from ischaemic heart disease and stroke attributable to higher-than-optimum blood glucose concentration: comparative risk assessment. Lancet. 2006; 368:1651–9. doi: 10.1016/S0140-6736(06)69700-6 17098083 10.1016/S0140-6736(06)69700-6

[9] Rubinstein AL, Irazola VE, Calandrelli M, Elorriaga N, Gutierrez L, Lanas F, et al Multiple cardiometabolic risk factors in the Southern Cone of Latin America: A population-based study in Argentina, Chile, and Uruguay. Int J Cardiol. 2015; 183:82–8. doi: 10.1016/j.ijcard.2015.01.062 Epub 2015 Jan 27. 25662056 10.1016/j.ijcard.2015.01.062 PMC4382451

[10] Rubinstein AL, Irazola VE, Poggio R, Bazzano L, Calandrelli M, Lanas Zanetti FT. Detection and follow-up of cardiovascular disease and risk factors in the Southern Cone of Latin America: the CESCAS I study. BMJ Open. 2011; 1:e000126 doi: 10.1136/bmjopen-2011-000126 22021769 10.1136/bmjopen-2011-000126 PMC3191438

[11] Craig CL, Marshall AL, Sjöström M, Bauman AE, Booth ML, Ainsworth BE, et al International physical activity questionnaire: 12-country reliability and validity. Med Sci Sports Exerc. 2003; 35(8):1381–95. doi: 10.1249/01.MSS.0000078924.61453.FB 12900694 10.1249/01.MSS.0000078924.61453.FB

[12] Guidelines for Data Processing and Analysis of the International Physical Activity Questionnaire (IPAQ). http://www.ipaq.ki.se/scoring.pdf. Accessed on April 10, 2014. 25376692

[13] Subar AF, Thompson FE, Kipnis V, Midthune D, Hurwitz P, McNutt S, et al Comparative validation of the Block, Willett, and National Cancer Institute food frequency questionnaires: the Eating at America's Table Study. Am J Epidemiol. 2001; 154(12):1089–99. 11744511 10.1093/aje/154.12.1089

[14] Elorriaga N, Irazola VE, Defago MD, et al Validation of a self-administered FFQ in adults in Argentina, Chile and Uruguay. Public Health Nutrition 2014 doi: 10.1017/S1368980013003431 24476763 10.1017/S1368980013003431 PMC10271131

[15] Friedewald WT, Levy RI, Fredrickson DS. Estimation of the concentration of low-density lipoprotein cholesterol in plasma, without use of the preparative ultracentrifuge. Clin Chem 1972; 18:499–502. 4337382

[16] World Health Organization Consultation. Definition, diagnosis and classification of diabetes mellitus and its complications, part 1: diagnosis and classification of diabetes mellitus. 1999 Geneva, World Health Organization.

[17] Plan and operation of the Third National Health and Nutrition Examination Survey, 1988–94. Series 1: programs and collection procedures. Vital Health Stat 1 1994; 32: 1–407. 7975354

[18] Argentina Census 2010. http://www.indec.gov.ar/nivel4_default.asp?id_tema_1=2&id_tema_2=41&id_tema_3=135 Last access October 1, 2016.

[19] Age standardization of rates: Anew WHO Standard. GPE Discussion Paper Series: No.31 EIP/GPE/EBD World Health Organization 2001.

[20] National Risk Factor Survey, Argentina, 2005. Ministerio de Salud de la Nación Argentina. http://www.msal.gob.ar/images/stories/bes/graficos/0000000553cnt-2014-10_encuesta-nacional-factores-riesgo-2005_informe-breve-final.pdf Last access October 1, 2016.

[21] National Risk Factor Survey, Argentina, 2009. Ministerio de Salud de la Nación Argentina. http://www.google.com.ar/url?sa=t&rct=j&q=&esrc=s&source=web&cd=4&ved=0CDQQFjADahUKEwi8167JnvDIAhVIiJAKHdgqBCY&url=http%3A%2F%2Fwww.bvs.org.ar%2Fpdf%2Fenfr2009.pdf&usg=AFQjCNEu5Eia_Lq7VGZ311Ku4UWlSq4H8A Last access October 1, 2016.

[22] National Risk Factor Survey, Argentina, 2013. Ministerio de Salud de la Nación Argentina. http://www.google.com.ar/url?sa=t&rct=j&q=&esrc=s&source=web&cd=1&ved=0CB0QFjAA&url=http%3A%2F%2Fwww.msal.gov.ar%2Fimages%2Fstories%2Fpublicaciones%2Fpdf%2F11.09.2014-tercer-encuentro-nacional-factores-riesgo.pdf&ei=aDpEVe_DIeKasQTNooHoCg&usg=AFQjCNEbeyGQqawlNqf8f1ntmkiMVgIOJw&bvm=bv.92291466,d.cWc Last access October 1, 2016.

[23] Ferrante D. National Risk Factors Survey 2009: evolution of the epidemic of chronic non communicable diseases in Argentina. Cross sectional study. Rev Argent Salud Pública 2011; 2: 34–41

[24] National Health Survey, Chile, 2010. Ministerio de Salud de Chile. http://www.google.com.ar/url?sa=t&rct=j&q=&esrc=s&source=web&cd=1&ved=0CCEQFjAAahUKEwiJi4KX2_HIAhWDQpAKHd2FDXs&url=http%3A%2F%2Fweb.minsal.cl%2Fportal%2Furl%2Fitem%2Fbcb03d7bc28b64dfe040010165012d23.pdf&usg=AFQjCNFPWCKaH7D4hhUv5yQ6Hc8ycVL_lA Last access October 1, 2016.

[25] National Risk Factor Survey, Uruguay, 2006. Ministerio de Salud de Uruguay. http://www.msp.gub.uy/sites/default/files/archivos_adjuntos/1er_enfrecnt_2006_1.pdf Last access October 1, 2016.

[26] National Risk Factor Survey, Uruguay, 2013. Ministerio de Salud de Uruguay. Preliminary results. http://www.msp.gub.uy/noticia/presentaci%C3%B3n-de-resultados-2%C2%AA-encuesta-nacional-de-factores-de-riesgo-de-enfermedades Last access October 1, 2016

[27] Escobedo J, Buitrón LV, Velasco MF, Ramírez JC, Hernández R, Macchia A, et al High prevalence of diabetes and impaired fasting glucose in urban Latin America: the CARMELA Study. Diabet Med. 2009; 9:864–71. doi: 10.1111/j.1464-5491.2009.02795.x 19719706 10.1111/j.1464-5491.2009.02795.x

[28] Schargrodsky H, Hernández-Hernández R, Champagne BM, Silva H, Vinueza R, Silva Ayçaguer LC, et al CARMELA: assessment of cardiovascular risk in seven Latin American cities. Am J Med. 2008; 1:58–65. doi: 10.1016/j.amjmed.2007.08.038 18187074 10.1016/j.amjmed.2007.08.038

[29] Pérez-Escamilla R, Villalpando S, Shamah-Levy T, Méndez-Gómez Humarán I. Household food insecurity, diabetes and hypertension among Mexican adults: results from Ensanut 2012. Salud Publica Mex. 2014;56 Suppl 1:s62–70. 25649455 10.21149/spm.v56s1.5167

[30] James C, Bullard KM, Rolka DB, Geiss LS, Williams DE, Cowie CC, Albright A, Gregg EW. Implications of alternative definitions of prediabetes for prevalence in U.S. adults. Diabetes Care. 2011; 2:387–91. doi: 10.2337/dc10-1314 21270196 10.2337/dc10-1314 PMC3024354

[31] Jahangir E, Irazola V, Rubinstein A. Need, enabling, predisposing, and behavioral determinants of access to preventative care in Argentina: analysis of the national survey of risk factors. PLoS One. 2012;7(9):e45053 doi: 10.1371/journal.pone.0045053 Epub 2012 Sep 12. 22984608 10.1371/journal.pone.0045053 PMC3440415

[32] Whiting D, Unwin N, Roglic G. Diabetes: equity and social determinants In Blas E, Kurup A, editors. *Equity*, *social determinants and public health programmes*. World Health Organization; 2010 p77–94.

[33] Linetzky B, De Maio F, Ferrante D, Konfino J, Boissonnet C. Sex-stratified socio-economic gradients in physical inactivity, obesity, and diabetes: evidence of short-term changes in Argentina. Int J Public Health. 2013; 58(2):277–84. doi: 10.1007/s00038-012-0371-z Epub 2012 May 22. 22615030 10.1007/s00038-012-0371-z

[34] Gerich J. Type 2 diabetes mellitus is associated with multiple cardiometabolic risk factors. Clin Cornerstone. 2007;8(3):53–68. 18452842 10.1016/s1098-3597(07)80028-7

[35] Carter P, Gray LJ, Troughton J, Khunti K, Davies MJ. Fruit and vegetable intake and incidence of type 2 diabetes mellitus: systematic review and meta-analysis. The BMJ. 2010;341:c4229 doi: 10.1136/bmj.c4229 20724400 10.1136/bmj.c4229 PMC2924474

[36] Morrish NJ, Stevens LK, Fuller JH, et al Risk factors for macrovascular disease in diabetes mellitus: the London follow-up to the SHO Multinational Study of Vascular Disease in Diabetes. Diabetologia. 1991;34:590–594. 1936663 10.1007/BF00400279

[37] Ziemer DC, Twombly JG, Phillips LS. Screening for diabetes and pre-diabetes with proposed A1C-based diagnostic criteria. Diabetes Care 2010;33:2184–2189. doi: 10.2337/dc10-0433 20639452 10.2337/dc10-0433 PMC2945158

[38] Mostafa SA, Khunti K, Srinivasan BT, Webb D, Gray LJ, Davies MJ. The potential impact and optimal cut-points of using glycated haemoglobin, HbA1C, to detect people with impaired glucose regulation in a UK multi-ethnic cohort. Diabetes Res Clin Pract 2010;90:100–108. doi: 10.1016/j.diabres.2010.06.008 20633944 10.1016/j.diabres.2010.06.008

[39] Kilpatrick ES, Bloomgarden ZT, Zimmet PZ. Is haemoglobin A1C a step forward for diagnosing diabetes? BMJ 2009;339:b4432 doi: 10.1136/bmj.b4432 19903702 10.1136/bmj.b4432

[40] Borch-Johnsen K, Colagiuri S. Diagnosing diabetes—time for a change? Diabetologia 2009;52:2247–2250. doi: 10.1007/s00125-009-1526-1 19756480 10.1007/s00125-009-1526-1

[41] Sikaris Ken. The Correlation of Hemoglobin A1c to Blood Glucose. J Diabetes Sci Technol 2009;3(3):429–438. doi: 10.1177/193229680900300305 20144279 10.1177/193229680900300305 PMC2769865

